# High prevalence of 19A pneumococcal serotype carriage during the COVID-19 pandemic in Brazil

**DOI:** 10.1016/j.bjid.2024.104467

**Published:** 2024-11-21

**Authors:** Muriel Primon-Barros, Fernanda Hammes Varela, Márcia Polese-Bonatto, Ivaine Tais Sauthier Sartor, Thais Raupp Azevedo, Caroline Nespolo de David, Maiko Luis Tonini, Renato T. Stein, Marcelo Comerlato Scotta, Cícero Armídio Gomes Dias

**Affiliations:** a Moinhos de Vento Hospital, Porto Alegre, RS, Brazil; b Universidade Federal de Ciências da Saúde de Porto Alegre (UFCSPA), Porto Alegre, RS, Brazil; c Pontifícia Universidade Católica do Rio Grande do Sul (PUC-RS), Escola de Medicina, Porto Alegre, RS, Brazil; d Coordenação Geral de Vigilância de Tuberculose, Micoses Endêmicas e Micobactérias Não Tuberculosas; Departamento de HIV/AIDS, Tuberculose, Hepatites Virais e IST/Secretaria de Vigilância em Saúde e Meio Ambiente; Ministério da Saúde (CGTM/DATHI/SVSA/MS), Brasília, DF, Brazil

**Keywords:** *Streptococcus pneumoniae*, 19A serotype, prevalence, pneumococcal conjugate vaccines, carriage

## Abstract

**Introduction:**

*Streptococcus pneumoniae* colonization patterns are influenced by host and environmental factors, which may be related to Invasive Pneumococcal Disease (IPD). Interestingly, COVID-19 pandemic witnessed a decline in the incidence of IPDs. Investigations with diligent data collection on the prevalence of nasopharyngeal colonization and associated serotypes during this unique period can yield novel insights. The aim of the current study was to assess the prevalence of *S. pneumoniae* carriage among children and adults who have sought care at emergency departments with suspected COVID-19.

**Methods:**

In this cross-sectional study, adults and children presenting with signs and symptoms likely associated with COVID-19 in two outpatient clinics in Southern Brazil were invited to participate. RT-PCR with a comprehensive molecular panel for pneumococcal identification of the 21 most prevalent serotypes in Latin America was performed on all enrolled subjects. Prevalence of pneumococcal carriage was assessed in the age groups (< 2, ≥ 2–5, ≥ 5–11, ≥ 11–18, ≥ 18–60, ≥ 60).

**Results:**

A total of 1644 subjects were included in the study. Pneumococcal carriage was detected by PCR testing in 14.9% (245/1,644), and serotype identification occurred in 42.0% (103/245) of the participants, with a total frequency of 111. The most frequent serotype identified was 19A (25.2%, n = 28/111), followed by 6C/6D (17.1%, n = 19/111), and 23A (11.7%, n = 13/111), also highlighting the high frequency of non-vaccine serotypes found across all age groups.

**Discussion:**

19A serotype, as well other most frequent serotypes identified are not covered by the PCV-10 in a community setting where PCV-10 is widely available. This finding reinforces the need for continuous surveillance to determine the impact of pneumococcal vaccination and guide public health decision-making. High 19A serotype prevalence is critical in the decision-making process for electing the best options for pneumococcal conjugate vaccines.

## Introduction

*Streptococcus pneumoniae* usually colonizes the human nasopharynx with variable carriage duration, most times for longer periods in children than adults, with carriage rates influenced by the environment, age of the affected population, and the presence/frequency of concomitant upper airway respiratory infections. A well-known finding is that colonization patterns are associated with invasive disease.^1^

Despite the introduction of Pneumococcal Conjugate Vaccines (PCVs), *S. pneumoniae* is still a significant pathogen in community-acquired pneumonia, and it is also associated with meningitis and sepsis in children, the elderly, and in high-risk populations.^2^, ^3^, ^4^, ^5^ Currently, over 100 pneumococcal serotypes have been identified, defined by their polysaccharide capsule, with variable invasive capabilities (case-carrier ratios); distribution of pathogenic isolates varies by time and geographical location of the surveys, age, site of infection, and immunization history of the study subjects.^6^

A change in the distribution of *S. pneumoniae* serotypes, with increased NVTs (especially serotypes 3, 6C, and 19A),^7^^,^^8^ has been described earlier in Brazil. Data on nasopharyngeal carriage and the distribution of pneumococcal serotypes among colonized children and adults are scarce in Brazil. PVC-10 (serotypes 1, 4, 5, 6B, 7F, 9V, 14, 18C, 19F, and 23F) was introduced in Brazil in 2010 through a free access National Immunization Program (NIP) to children under two years old,^9^ since 2011 the vaccine coverage in children varied between 81.65% and 95.25%.^10^ The introduction of PCV-10 was associated with a significant decrease in the incidence of pneumonia and IPD in both vaccinated and unvaccinated cohorts. PCV-13 (which includes PVC-10 serotypes, plus serotypes 3, 6A, and 19A) has been available in the private sector in Brazil since 2009^11^ but has yet to be incorporated into the public system. It is important to note that new pneumococcal conjugate vaccines, such as PCV-15 (PCV-13 serotypes, plus serotypes 22F and 33F) and PCV-20 (PVC-15 serotypes, plus serotypes 8, 10A, 11A, 12F, and 15B) have recently been approved for use both for children and adults in some countries.^12^^,^^13^

Social restrictions in response to the COVID-19 pandemic offered a unique opportunity to learn about the dynamics of transmission of infectious agents. During the COVID-19 pandemic, with its social isolation measures, there was a decrease in the incidence of invasive pneumococcal disease observed globally.^14^^,^^15^ However, little is known about pneumococcal colonization patterns and their serotypes among carriers during that period.

The main aim of this study is to describe the prevalence of pneumococcal colonization in children and adults with signs and symptoms of COVID-19-like illness prospectively enrolled in a cross-sectional study during the pandemic. We have also assessed pneumococcal serotypes by age group in relation to their immunization status. The potential impact of current and new conjugate vaccines is also discussed here.

## Materials and methods

### Study design

A cross-sectional study was done from May to November 2020, adults and children (≥ 1 month old) seeking care with symptoms suggestive of COVID-19 (fever, and/or cough, and/or sore throat within 14 days of onset) were consecutively enrolled in two Emergency Rooms, in Porto Alegre City, Southern Brazil (Hospital Moinhos de Vento, a private tertiary hospital with 372 general wards and 113 Intensive Care Unit (ICU) beds and Hospital Restinga e Extremo Sul, a public secondary hospital with 485 beds). The failure to properly collect nasopharyngeal samples was the main exclusion criterion. At enrollment, demographic and clinical data, signs, and symptoms were collected. For children under 11 years, pneumococcal vaccination status regarding PCV-10 and PCV-13 was assessed at enrollment (a questionnaire was applied to legal guardians, and the answers were self-reported). The prevalence of pneumococcal was performed according to the age groups (< 2 years; ≥ 2–5 years; ≥ 5–11 years; ≥ 11–18 yr; ≥ 18–60 years; ≥ 60 years). We also performed a sub-analysis stratified by age, considering eligibility criteria for vaccination and the representativeness of the pneumococcal prevalence according to these groups. PCV-10 entered Brazilian NIP in 2010 for all children < 2 years and some with specific comorbidities < 5 years. Therefore, in 2020, the children covered by PCV-10 would be up to those with 11 years-old. The 6C/6D, 15A/15F, 16F, and 23A were considered non-vaccine serotypes since they are not presented in any approved conjugate vaccine.^13^

### Sample collection and respiratory pathogens detection

All children collected samples with a bilateral nasal swab, stored at −80 °C until the end of all inclusions, and were analyzed by RT-PCR. The presence of *Streptococcus pneumoniae; Bordetella pertussis; Chlamydophila pneumoniae; Mycoplasma pneumoniae*; adenovirus; bocavirus; coronavirus types HKU1, 229E, NL63, and OC43; influenza A virus types H1 and H3; influenza B virus; human enterovirus; human metapneumovirus; parainfluenza virus types 1, 2, and 3; Respiratory Syncytial Virus (RSV) types A and B; rhinovirus, and SARS-CoV-2 was evaluated according to.^16^ The analyses were performed at the Molecular Biology Research Laboratory at Hospital Moinhos de Vento.

### Streptococcus pneumoniae detection and serotyping

RNA for pneumococcal molecular detection was extracted using MagMax™ Viral/Pathogenic Nucleic Acid Isolation (Applied Biosystems) in the KingFisher Duo Prime. RNA dilutions (between 0.5 and 2.0 ng/μL) were prepared from the previous quantification using NanoDrop™ Lite Spectrophotometer (ThermoFisher, USA) and added in the RT-PCR assay with the Path™ 1-Step RT-qPCR System platform Master Mix CG (Applied Biosystems, USA), TaqMan® Microbial Assays-single tube assay (Applied Biosystems, USA) with the Ba06439619_s1 probe for the *S. pneumoniae* target (Applied Biosystems, USA). A control reaction was performed with TaqMan® Respiratory Tract Microbiota Amplification Control (Thermofisher, USA). DNA extraction for pneumococcal serotyping was done as per the Centers for Disease Control and Prevention (CDC) protocol^17^ using the QIAampDNA Mini kit or DNeasyBlood & Tissue Kit (Qiagen, Germany). Amplification of the *lytA* gene (F-CAGCGGTTGAACTGATTGA and R-TGGTTGGTTATTCGTGCAA primers) was confirmed by RT-PCR using Promega GoTaq™ qPCR Master Mix (Promega, USA).^18^ *S. pneumoniae* colonization was considered when the *lytA* gene was identified. The *S. pneumoniae* serotyping followed the CDC protocol for triplex sequential real-time PCR-serotyping Latin America,^17^^,^^19^ testing the 21 serotypes more prevalent in Latin America (1, 2, 3, 4, 5, 6A/6B/6C/6D, 6C/6D, 7F/7A, 9V/9A, 11A/11D, 12F/12A/12B/44/46, 14, 15A/15F, 16F, 18C/18F/18B/18A, 19A, 19F, 22F/22A, 23A, 23F, and 33F/33A/37) through TaqMan^TM^ fast advanced Master Mix (Applied Biosystems, USA). Serotypes 6A and 6B were differentiated using specific primers previously described.^20^

### Statistical analysis

Data normality assumptions were verified for continuous variables, the Mann-Whitney-Wilcoxon test was applied, and values were presented as median and Interquartile Ranges (IQR). Categorical variables were described as percentages, and Pearson's Chi-Square test or Fisher's Exact test were used to evaluate the association of clinical and demographic data between all individuals and for those with < 11 and ≥ 11 years-old colonized or non-colonized by *S. pneumoniae*. The same comparisons were also performed to evaluate the association of pneumococcal serotypes among age groups (children: < 2 years, ≥ 2–5 years, ≥ 5–11 years; and teenagers and adults: < 18 years, ≥ 18–60 years, ≥ 60 years). The frequency of serotypes was also analyzed according to the coverage of all the pneumococcal conjugate vaccines approved (10, 13, 15, and 20-valent). All data preprocessing and analyses were performed in R 4.1.1 statistical software^21^ using the plyr, dplyr, and ggplot2 libraries.

### Ethics

The Hospital Moinhos de Vento Institutional Review Board (IRB n° 4.637.933) approved the study. The Good Clinical Practice Guidelines and decree 466/12 of the Brazilian National Health Council^22^ were followed. All participants in this study provided written informed consent, and legal guardians provided signed consent and authorization for children's participation in the study.

## Results

**Fig. 1:**
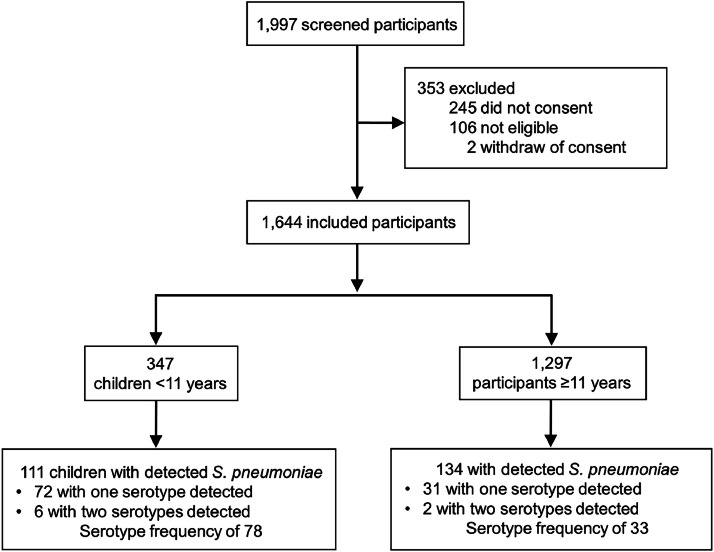
Subjects flowchart. Total 1997 subjects were screened, and 353 were excluded. A total of 1644 participants were included in the study. Of this, 347 were children < 11 years (eligible for pneumococcal vaccination), and 1297 were ≥ 11 years (not-eligible for pneumococcal immunization).

A total of 1997 subjects were screened, and 353 were excluded (245 for not consenting, 106 for not meeting inclusion criteria, and 2 for withdrawing consent) (Fig. 1). Total 1644 subjects were enrolled in the study with a median age of 32.1 years (IQR 15.7‒43.7, range 0.2‒99.9). Pneumococcal carriage was detected in 14.9% (245/1644) subjects, and the prevalence of *S. pneumoniae* carriage according to age group was: 39.4% (43/109) in children < 2 years of age; 34.3% (34/99) in children aged 2–5 years; 24.5% (34/139) for those between 5‒11 years; 15.7% (14/89) for those between 11‒18 years; 10.3% (111/1078) for those between 18‒60yr; and 6.9% (9/130) for those ≥ 60. Serotypes were identified in 103 participants (42.0%, 103/245), with 95 individuals with one single serotype identified, and 8 individuals with two serotypes identified (19A and 3; 19A and 7F/7A; 11A/11D and 23A; 5 and 33F/33A/37; 19A and 6C/6D; 19A and 33F/33A/37; 19A and 14; 15A/15F 15A/15F and 33F/33A/37), with a total frequency of 111 times that the serotypes were found.

**Fig. 2:**
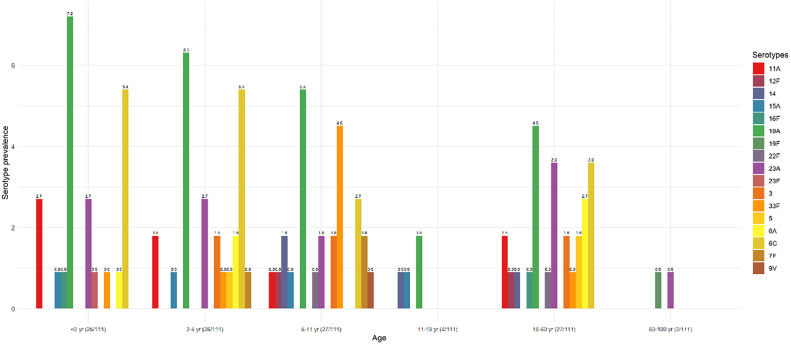
Prevalence of the pneumococcal serotype by age group. Data in brackets in the X-axis label shows the number of serotypes according to the age group by the total number of identified serotypes.

The most frequent serotype was 19A (25.2%, n = 28/111), followed by 6C/6D (17.1%, n = 19/111), and 23A (11.7%, n = 13/111). The serotype distribution according to age groups is shown in Fig. 2. The serotype prevalence achieved in children < 2 years of age was 22.5% (25/111), while this was 23.4% among children aged 2‒5 years (26/111), 24.3% for those between 5‒11 years (27/111), 3.6% for those between 11‒18 years (4/111), 24.3% for those between 18–60 year (27/111), and 1.8% for those ≥ 60 (2/111).

**Fig. 3:**
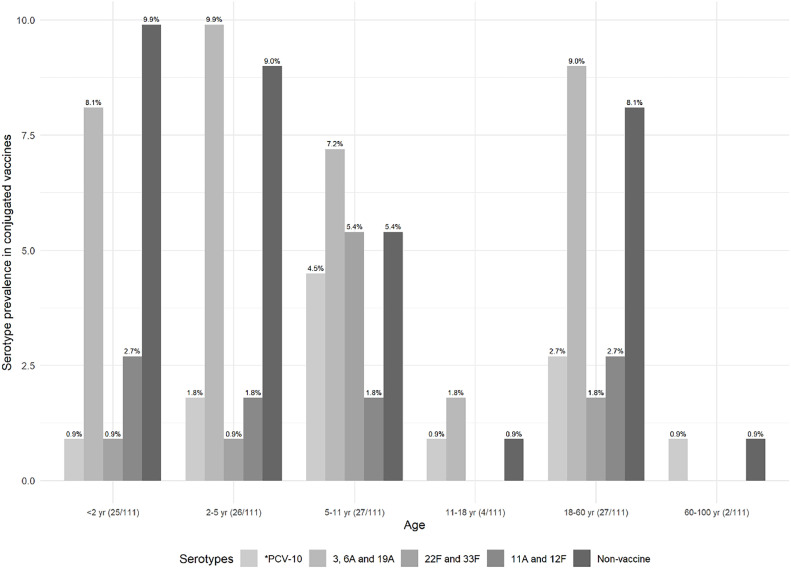
Prevalence of the pneumococcal serotype in the conjugated vaccines according to the age group. *PCV-10 comprises 1, 4, 5, 6B, 7F/7A, 9V/9A, 14, 18C/18F/18B/18A, 19F, and 23F serotypes. Exclusive serotypes for PCV-13 comprise 3, 6A,19A, for PCV-15 comprise 22F and 33F, and for PCV-20 comprise 8, 10A, 11A, 12F and 15B Non-vaccine serotypes include 6C/6D, 15A/15F, 16F, and 23A. Non-vaccine comprises 6C/6D, 15A/15F, 16F, and 23A serotypes. Parenthesis in the x-axis label shows the number of the identified serotypes by age group, considering the total number of identified serotypes.

Prevalence of *S. pneumoniae* serotypes in each age group, based on the serotypes present in the conjugated vaccine, is shown in Fig. 3.

A total of 347 enrolled subjects were classified as vaccine-eligible population (children < 11 years), with a median age of 3.6 years (IQR 1.5‒7.2). The remaining 1297 were 11 years or older, with a median age of 36.8 years (IQR 27.5‒47.3), as shown in Table 1.

**Table 1:** Demographic and clinical data of participants eligible for vaccine (up to 11-years-old) and not-eligible (≥ 11-years-old).

	Vaccine eligible (< 11-years-old)			Vaccine not‒eligible (≥ 11-years-old)		
Characteristics	Total (n = 347)	*S. pneumoniae* positive (n = 111)	*S. pneumoniae* negative (n = 236)	Total (n = 1.297)	*S. pneumoniae* positive (n = 134)	*S. pneumoniae* negative (n = 1162)
Age, median (IQR, range)	3.57 (1.48‒7.23, 0.18‒10.93)	2.72 (1.25‒5.72, 0.19‒10.64)	4.20 (1.71‒8.02, 0.18‒10.93)	36.79 (27.52‒47.32, 11.09‒99.86)	35.81 (25.45‒43.65, 12.37‒78.68)	36.84 (27.74‒47.70, 11.09‒99.86)
Female sex, n (%)	185 (53.31)	50 (45.05)	135 (57.20)	775 (59.75)	85 (63.43)	689 (59.29)
Pneumococcal vaccination						
Yes, n (%)	298 (85.88)	101 (90.99)	197 (83.47)	‒	‒	‒
No, n (%)	45 (12.97)	10 (9.01)	35 (14.83)	‒	‒	‒
NA, n (%)	4 (1.15)	0 (0.00)	4 (1.69)	‒	‒	‒
Type of pneumococcal vaccine reported						
Yes (PCV-10), n (%)	245/298 (82.21)	91 (81.89)	154 (65.25)	‒	‒	‒
Yes (PCV-13), n (%)	53/298 (17.79)	10 (9.01)	43 (18.22)	‒	‒	‒

### Vaccine-eligible population (< 11yr)

*Streptococcus pneumoniae* was detected in 111/347 (32.0%) children (median age 2.7 years, IQR 1.2‒5.7). From the 111 participants with *S. pneumoniae* detection, the serotyping identification occurred in 64.9% of subjects (72/111), and 8.3% (6/72) had more than one identified serotype (Fig. 1). In 298 out of 347 children (85.9%) were vaccinated against *S. pneumoniae*, as informed by their caregivers.

The frequency of pneumococcal serotypes was 78 in this population; the most prevalent one was 19A (18.9%, 21/78), followed by 6C/6D (13.5%, 15/78), as shown in Table 2. The non-vaccine serotypes were seen in 34.6%, and non-identification occurred in 29.7% (33/111) of them. The serotypes identified according to vaccine coverage and prevalence according to the age groups are detailed in Table 2.

**Table 2:** Identified serotypes in the population eligible for vaccine (up to 11 years old) and not eligible (≥ 11 years old).

	Vaccine eligible (< 11-years-old)	Vaccine not-eligible (≥ 11-years-old)
Serotypes according to conjugated vaccines (n = 111)	Identified serotypes (n = 78)	Identified serotypes (n = 33)
**PCV-10 serotypes, n (%)**		
1	‒	‒
4	‒	‒
5	1 (1.28)	2 (6.06)
6B	‒	‒
7F^a^	3 (3.85)	‒
9V^a^	1 (1.28)	‒
14	2 (2.56)	2 (6.06)
18C	‒	‒
19F	‒	1 (3.03)
23F	1 (1.28)	‒
Total	8 (10.26)	5 (15.15)
**PCV-13 serotypes, n (%)**		
3	4 (5.13)	2 (6.06)
6A^a^	3 (3.85)	3 (9.09)
19A	21 (26.92)	7 (21.21)
Total	28 (35.90)	12 (36.36)
**PCV-15 serotypes, n (%)**		
22F^a^	1 (1.28)	1 (3.03)
33F^a^	7 (8.97)	1 (3.03)
Total	8 (10.26)	2 (6.06)
**PCV-20 serotypes, n (%)**		
8	‒	‒
10A	‒	‒
11A^a^	6 (7.69)	2 (6.06)
12F^a^	1 (1.28)	1 (3.03)
15B	‒	‒
Total	7 (8.97)	3 (9.09)
**Non-vaccine serotypes, n (%)**		
6C/6D	15 (19.23)	4 (12.12)
15A/15F	3 (3.85)	1 (3.03)
16F	1 (1.28)	1 (3.03)
23A	8 (10.26)	5 (15.15)
Total	27 (34.62)	11 (33.33)

^a^The corresponding vaccine refers to this specific serotype; however, we identified the 7F/7A, 9V/9A, 22F/22A, 33F/33A/37, 11A/11D, and 12F/12A/12B/44/46 serotypes according to the CDC 2021 protocol for Latin America *S. pneumoniae* PCR-serotyping.^17^^,^^19^

Interestingly, there was a significant association between the independent detection of both rhinovirus and adenovirus with *S. pneumoniae* colonization (p < 0.001 and p = 0.014, respectively), as shown in Supplementary Table 1. There was no significant association between *S. pneumoniae* colonization and infection with SARS-CoV-2, enterovirus, coronavirus NL63, metapneumovirus, *Mycoplasma pneumoniae*, or *Chlamydophyla pneumoniae*. There were no positive samples for *Bordetella pertussis*, bocavirus, coronavirus types HKU1, 229E, and OC43, influenza A virus types H1 and H3, influenza B virus, parainfluenza virus types 1, 2, and 3, or RSV types A and B in this group of children.

### Not-eligible vaccine group (children ≥ 11 year and adults)

134/1.297 (10.3%) subjects were colonized by *S. pneumoniae*, with the same pneumococcal serotyping profile observed in children. From the 134 participants with *S. pneumoniae* detection, the serotyping identification occurred in 23.1% of subjects (31/134), and 6.5% (2/31) had more than one identified serotype (Fig. 1). The frequency of pneumococcal serotypes was the frequency of pneumococcal serotypes was 33, and the 19A also was the most common (21.2%, 7/33). The non-vaccine serotypes (6C/6D, 15A/15F, 16F, and 23A) were seen in 33.3% (11/33) of them (Table 2). There was no detection of *Bordetella pertussis*, bocavirus, coronavirus types 229E and OC43, influenza A virus types H1 and H3, influenza B virus, parainfluenza virus types 1, 2, and 3, or RSV types A and B among this group of subjects (Supplementary Table 2).

## Discussion

The prevalence of pneumococcal colonization among all age groups seems to be comparable to the findings observed prior to the pandemic.^23^, ^24^, ^25^ Despite an observable reduction in the incidence of invasive pneumococcal disease, which was extensively described during the COVID-19 pandemic, our findings suggest that *S. pneumoniae* colonization was not impacted in the same way, as shown in Israel.^26^^,^^27^ This discrepancy between pneumococcal colonization and significantly lower frequencies of severe disease points toward a more important role for respiratory viruses as drivers of pneumococcal invasion. Many studies show that viral transmission (especially RSV and influenza) during this period was markedly reduced.^28^, ^29^, ^30^

We have also detected a high prevalence of the 19A serotype at all ages in a setting where PCV-10 is widely available for children in the Brazilian NIP. Data from the National Reference Laboratory (NRL) of the Brazilian Ministry of Health registered 360 events of bacterial meningitis and invasive pneumococcal diseases in 2020 (same year that the samples were collected), of which 71 (19.7%) were 19A serotypes in all age groups described.^31^ In the same line, the high frequency of exclusively PCV-13 serotypes (3, 6A, and 19A) was predominantly identified among this study population (35.9% in < 11 years, and 36.4% ≥ 11 years). Interestingly, although we found a colonization rate comparable to the pre-pandemic period, there was a decrease in bacterial meningitis and pneumococcal disease events (360 events in 2020) compared to the pre-pandemic period (890 events in 2019).^31^

As expected, a low prevalence of PCV-10 serotypes (1, 4, 5, 6B, 7F, 9V, 14, 18C, 19F, and 23F) was observed after ten continuous years of vaccine availability. Beyond a marked reduction in the prevalence of PCV-10 serotypes causing invasive disease after PCV10 introduction, reductions greater than 90% in the prevalence of such serotypes were also reported in nasopharyngeal colonization among children when pre and post vaccination periods were compared in Brazil, reinforcing the high effectiveness of pneumococcal conjugate vaccines against pneumococcal nasopharyngeal carriage.^8^^,^^32^ Even though the PCV-10 vaccine was not intended for the adult population, the array of positive serotypes resembled that of children, possibly due to the reduced transmission of vaccine-type strains. These findings underscore the significant protective herd immunity concept, likely resulting from successfully vaccinating young children with PCV-10.^33^, ^34^, ^35^ Our findings suggest reinforcement of both the effectiveness of pneumococcal conjugate vaccines in reducing specific pneumococcal serotypes’ carriage in the nasopharyngeal, and the fact that the most frequent serotype found was 19A, not yet contemplated in the Brazilian National Immunization Program vaccine. Highlighting that active surveillance systems are in place and necessary, especially when vaccines with broader pneumococcal coverage are being introduced or have not yet been introduced.

One of our key findings is the high frequency of detected non-vaccine serotypes (higher than 33.3% at all ages), even if one considers a complete set of serotypes, including the new 20-valent pneumococcal conjugate vaccine. Even if not all these serotypes have the same level of invasiveness, this information should not go unnoticed. After five years of the PCV-10 introduction in Brazil, the 19A, 6C, and 3 (non-PCV10 types) were already the major causes of IPD in the country, with a predominance of the 19A serotype, which has been distinctly detected as the leading cause of meningitis and the second cause of non-meningitis invasive episodes in all age groups.^8^

It is important to compare the range of non-vaccine serotypes (6C/6D, 15A/15F, 16F, and 23A) found in our study with reported cases by the Brazilian NRL: 52 events of IPD for serotypes 6C/6D (n = 26), 15A/15F (n = 10), 16F (n = 6), and 23A (n = 10), which represent 14.4% of the total of 360 cases registered in the country, in 2020.^31^ These non-vaccine serotypes' colonization and invasiveness potential can be considered for future vaccine updates.

The present study has some limitations. First, the information about the PCV-10 and PCV-13 vaccination is self-reported, and it was not possible to confirm in vaccination cards which of these vaccines were really administered. Additionally, as PCV-10 and PCV-13 entered the public and private systems, respectively in 2010, the number of people over 11-years of age vaccinated was negligible. Second, due to the way samples were collected and stored, it was not possible to conduct the investigation with methods based on bacteriological culture. However, this problem was circumvented by using well-established molecular techniques, and the prevalence of colonization found in both children and adults is in line with previous findings.^23^^,^^24^ Third, only 21 probes were used to identify the most prevalent serotypes in Latin America, which may reflect higher rates of no serotyping (29.7% in children under 11 years old and 75.4% in participants ≥11 years-old); we have followed the CDC 2021 protocol for *S. pneumoniae* PCR-serotyping for Latin America, which in some cases, does not differentiate one specific serotype, but a similar set, and because of this, after our research done, this previous triplex scheme has been updated by CDC to a new expanded sequential real-time PCR scheme, with 12 quadriplex reactions for detection of 64 serotypes as individual serotypes or small serogroups.^19^ Last, as colonization was investigated in individuals with signs and symptoms of COVID-19, it might impact the microbiome.

## Conclusions

In our population, the prevalence of pneumococcal nasopharyngeal carriage in participants suspected of having COVID-19 appears to be similar to pre-pandemic findings. We have also detected a high frequency of 19A, followed by 6C/6D, and 23A pneumococcal serotypes in the community. Although we all have positive expectations with the forthcoming of recently approved pneumococcal conjugate vaccines, rates of non-vaccine serotypes, as detected in this study, indicate (even with different degrees of invasiveness ability) that we should pay close attention to the patterns of serotype prevalence and distribution in this new environment. Specially, the high 19A serotype prevalence is critical in the decision-making process for electing the best options for pneumococcal conjugate vaccines.

## Funding

This work was supported by the Brazilian Ministry of Health through the Institutional Development Program of the Brazilian National Health System (PROADI-SUS) in collaboration with Hospital Moinhos de Vento.

## Conflicts of interest

The authors declare no conflicts of interest.

## Appendix Supplementary materials

Image, application 1
