# Supplementary material for: High prevalence of 19A pneumococcal serotype carriage during the COVID-19 pandemic in Brazil
Source: Braz J Infect Dis. 2024 Nov 21;28(6):104467. doi: 10.1016/j.bjid.2024.104467 (PMC11616489; doi:10.1016/j.bjid.2024.104467)
Supplement: Supplementary file 1 [file mmc1.docx]

**BJID-D-24-00130_Supplementary Material**

**Supplementary Table 1** Pathogen detection and the pneumococcal serotypes of vaccine-eligible children (up to 11-years).

| **Vaccine eligible children (< 11-years-old)** | | | | |
| --- | --- | --- | --- | --- |
|  | **Total**  **(n = 347)** | ***S. pneumoniae* positive**  **(n = 111)** | ***S. pneumoniae* negative**  **(n = 236)** | **p-value** |
| **Detected pathogens, n (%)** |  |  |  |  |
| Rhinovirus | 181/346 (52.31) | **78 (70.27)** | 103 (43.64) | **<0.001^a^** |
| SARS-CoV-2 | 59 (17.00) | 16 (14.41) | 43 (18.22) | 0.467^a^ |
| Enterovirus | 7/346 (2.02) | 3 (2.70) | 4 (1.69) | 0.685^b^ |
| Adenovirus | 6/346 (1.73) | **5 (4.50)** | 1 (0.42) | **0.014^b^** |
| *Mycoplasma pneumoniae* | 6/346 (1.73) | 3 (2.70) | 3 (1.27) | 0.390^b^ |
| Coronavirus NL63 | 2/346 (0.58) | 2 (1.80) | 0 (0.00) | 0.102^b^ |
| Metapneumovirus | 2/346 (0.58) | 1 (0.90) | 1 (0.42) | 0.539^b^ |
| *Chlamydophila pneumoniae* | 1/346 (0.29) | 1 (0.90) | 0 (0.00) | 0.321^b^ |
| **Racial or ethnic group, n (%)** |  |  |  |  |
| Caucasian | 235/331 (71.00) | 62 (55.86) | **173 (73.31)** | **0.009^a^** |
| Non-caucasian | 96/331 (29.00) | 40 (36.04) | 56 (23.73) |  |

* Mann-Whitney-Wilcoxon test.

^a^ Pearson's Chi-Squared test.

^b^ Fisher's exact test.

**Supplementary Table 2** Pathogen detection and the pneumococcal serotypes of vaccine not-eligible participants (≥ 11-years-old).

| **Vaccine not-eligible participants (≥ 11-years-old)** | | | | |
| --- | --- | --- | --- | --- |
|  | **Total**  **(n = 1,297)** | ***S. pneumoniae* positive**  **(n = 134)** | ***S. pneumoniae* negative**  **(n = 1,162)** | **p-value** |
| **Detected pathogens, n (%)** |  |  |  |  |
| SARS-CoV-2 | 472/1296 (36.42) | 42 (31.34) | 430 (37.01) | 0.229^a^ |
| Rhinovirus | 449/1291 (34.78) | **75 (55.97)** | 373 (32.10) | **<0.001^a^** |
| *Mycoplasma pneumoniae* | 18/1291 (1.39) | 4 (2.99) | 14 (1.20) | 0.108^b^ |
| Coronavirus HKU1 | 9/1291 (0.70) | 0 (0.00) | 9 (0.77) | 0.610^b^ |
| Enterovirus | 7/1291 (0.54) | 1 (0.75) | 6 (0.52) | 0.537^b^ |
| Metapneumovirus | 4/1291 (0.31) | 2 (1.49) | 2 (0.17) | 0.056^b^ |
| Adenovirus | 3/1291 (0.23) | 1 (0.75) | 2 (0.17) | 0.281^b^ |
| Coronavirus NL63 | 2/1291 (0.15) | 0 (0.00) | 2 (0.17) | 1.000^b^ |
| **Racial or ethnic group, n (%)** |  |  |  |  |
| Caucasian | 897 (74.63) | 92 (68.66) | 804 (69.19) | 0.998^a^ |
| Non-caucasian | 305 (25.37) | 32 (23.88) | 273 (23.49) |  |

* Mann-Whitney-Wilcoxon test.

^a^ Pearson's Chi-Squared test.

^b^ Fisher's exact test.
